# Revision of the genus *Ptomaphagus* Hellwig (Coleoptera, Leiodidae, Cholevinae) from Japan

**DOI:** 10.3897/zookeys.607.9074

**Published:** 2016-07-28

**Authors:** Cheng-Bin Wang, Jan Růžička, Masaaki Nishikawa, Michel Perreau, Yasuhiko Hayashi

**Affiliations:** 1 Department of Ecology, Faculty of Environmental Sciences, Czech University of Life Sciences Prague, Kamýcká 129, CZ-165 21 Praha 6, Czech Republic; 2 Kashiwagaya 1112–16, Ebina, 243–0402 Japan; 3 IUT Paris Diderot, Université Paris Diderot case 7139 Sorbonne Paris Cité, 5, rue Thomas Mann, 75205 Paris cedex 13; 4 Suimeidai 3-1-73, Kawanishi, 666-0116 Japan

**Keywords:** Leiodidae, Cholevinae, *Ptomaphagus*, taxonomy, new species, new synonyms, Japan

## Abstract

After examining Japanese material of *Ptomaphagus* Hellwig from various collections, a new species is described, Ptomaphagus (s. str.) piccoloi **sp. n.**, and a new subjective synonym proposed, Ptomaphagus (s. str.) kuntzeni Sokolowski, 1957 = Ptomaphagus (s. str.) amamianus Nakane, 1963, **syn. n.**, in this paper. Relevant morphological characters of examined species of *Ptomaphagus* are illustrated with colour plates, and known distributions are mapped.

## Introduction

*Ptomaphagus* Hellwig, 1795 is the most speciose genus (including 136 known species) in the tribe Ptomaphagini (Coleoptera, Leiodidae, Cholevinae). However, the nominotypical subgenus, which is limited to the Palaearctic and north Oriental regions has only 28 species (Perreau 2000, Nishikawa 2011).

In the fauna of Japan, only three species in the subgenus *Ptomaphagus* s. str. had been recorded before this study, namely Ptomaphagus (s. str.) sibiricus Jeannel, 1934, Ptomaphagus (s. str.) kuntzeni Sokolowski, 1957 and Ptomaphagus (s. str.) amamianus Nakane, 1963.

However, when we examined specimens previously identified as Ptomaphagus (s. str.) kuntzeni and Ptomaphagus (s. str.) amamianus from various collections, we found there are no differences at the specific level between them. After examining both holotypes, a new subjective synonym is proposed: Ptomaphagus (s. str.) kuntzeni Sokolowski, 1957 = Ptomaphagus (s. str.) amamianus Nakane, 1963, syn. n. Moreover, examined specimens previously identified as Ptomaphagus (s. str.) sibiricus from Japan are conspicuously different to the holotype of Ptomaphagus (s. str.) sibiricus which was described from Vladivostok, Russia (Jeannel 1934). Therefore, a new species from Japan is described and illustrated here: Ptomaphagus (s. str.) piccoloi sp. n. The dubious occurrences of Ptomaphagus (s. str.) kuntzeni in Myanmar and Ptomaphagus (s. str.) sibiricus in Japan, as well as several new island records, and the bionomics of the two species are briefly discussed in this paper. Relevant morphological characters of examined species of *Ptomaphagus* are illustrated with colour plates, and known distributions are mapped.

## Material and methods

Specimens were relaxed and softened in a hot saturated solution of potassium hydroxide for 4 minutes (for mounted dry specimens) or 8 minutes (for alcohol-preserved specimens), and then transferred to distilled water to rinse the residual potassium hydroxide off and stop any further bleaching. The softened specimens were moved into glycerin and dissected there to observe morphological details. After examination, the body parts were mounted on a glass slip with Euparal Mounting Medium for future studies. Habitus photographs were taken using a Canon macro photo lens MP-E 65mm on a Canon 550D. Observations, photographs and measurements of morphological details were performed using an Axio Zoom.V16 motorized stereo zoom microscope with an AxioCam MRc 5 in Beijing, or an Olympus BX53 microscope with an Olympus DP73 in Prague. The final deep focus images were created with Helicon Focus 5.3 stacking software in Beijing or Zerene Stacker 1.04 in Prague. The program Adobe Photoshop CS6 was used for post processing. Exact label data are cited for all specimens examined. Authors’ remarks and addenda are placed in square brackets, separate label lines are indicated by a slash (/) and separate labels by a double slash (//). Measurements are mean values based on 5 specimens.

The material examined for this study is deposited in the following collections and museums:

**BMNH** Natural History Museum (formerly British Museum), London, United Kingdom (M. Barclay)

**CCBW** Collection of Cheng-Bin Wang, Chengdu, China

**CHHF** Collection of Hideto Hoshina, Fukui University, Fukui, Japan

**CJRZ** Collection of Jan Růžička, Prague, Czech Republic

**CMNE** Collection of Masaaki Nishikawa, Ebina, Japan

**CMPR** Collection of Michel Perreau, Paris, France

**CPJA** Collection of Paweł Jałoszyński, Wrocław, Poland

**CYFO** Collection of Yoshifumi Fujitani, Okayama, Japan

**CYHK** Collection of Yasuhiko Hayashi, Kawanishi, Japan

**EUM** Ehime University Museum, Matsuyama, Japan (H. Yoshitomi)

**HUM** Hokkaido University Museum, Sapporo, Japan (M. Ôhara)

**MHNG** Muséum d’Histoire Naturelle, Genève, Switzerland (G. Cuccodoro)

**MNHA** Museum of Nature and Human Activities, Hyôgo, Japan (T. Yamauchi)

**NMPC** Národní museum, Prague, Czech Republic (J. Hájek)

**NSMT** National Museum of Nature and Science, Tsukuba, Japan (S. Nomura)

**SDEI** Senckenberg Deutsches Entomologisches Institut, Müncheberg, Germany (L. Behne)

**ZMHB** Museum für Naturkunde – Leibniz-Institut für Evolutions- und Biodiversitätsforschung an der Humboldt-Universität zu Berlin, Berlin, Germany (J. Frisch).

The following abbreviations are used for the measurements in millimetres (mm):

**AL** (antennal length): length from the antennal base to apex.

**BTW** (basitarsal width): maximum width of 1st protarsomere.

**EBL** (extended body length): summation of HL, PL, ELL, and length of exposed scutellum, preventing the error introduced by exposed or retracted head.

**ELL** (elytral length): length from the tail end of scutellum to the elytral apex.

**ELW** (elytral width): maximum width of two elytra combined together.

**EW** (eye width): width of a single compound eye in dorsal view.

**HL** (head length): axial length from the anterior apex of clypeus through the posterior margin of occipital carina.

**HW** (head width): maximum width of head (usually including eyes).

**PL** (pronotal length): axial length of the pronotum.

**PW** (pronotal width): maximum width of pronotum.

**TW** (tibial width): maximum width of protibia (excluding spines along outer margin etc.).

## Taxonomy

### Genus Ptomaphagus

Taxon classificationAnimaliaColeopteraLeiodidae

Hellwig, 1795

#### Distribution.

Holarctic, north Oriental, north Neotropical.

### Subgenus Ptomaphagus

Taxon classificationAnimaliaColeopteraLeiodidae

s. str.

#### Distribution.

Palaearctic, north Oriental.

### Ptomaphagus (s. str.) kuntzeni

Taxon classificationAnimaliaColeopteraLeiodidae

Sokolowski, 1957

**Figure 1.**
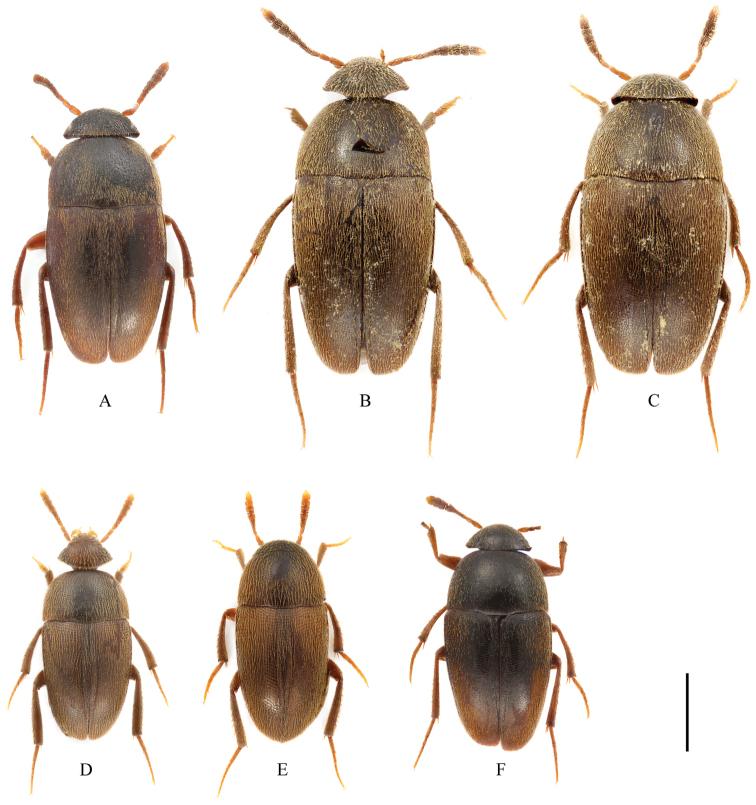
Habitus of *Ptomaphagus* (s. str.) spp. (dorsal view). **A** Ptomaphagus (s. str.) kuntzeni Sokolowski, 1957 ♂ (holotype) **B** Ptomaphagus (s. str.) amamianus Nakane, 1963, syn. n. ♂ (paratype) **C** Ptomaphagus (s. str.) amamianus Nakane, 1963, syn. n. ♀ (paratype) **D** Ptomaphagus (s. str.) piccoloi sp. n. ♂ (holotype) **E** Ptomaphagus (s. str.) piccoloi sp. n. ♀ (paratype) **F** Ptomaphagus (s. str.) sibiricus Jeannel, 1934 ♀ (holotype). Scale bar 1 mm.

**Figure 2.**
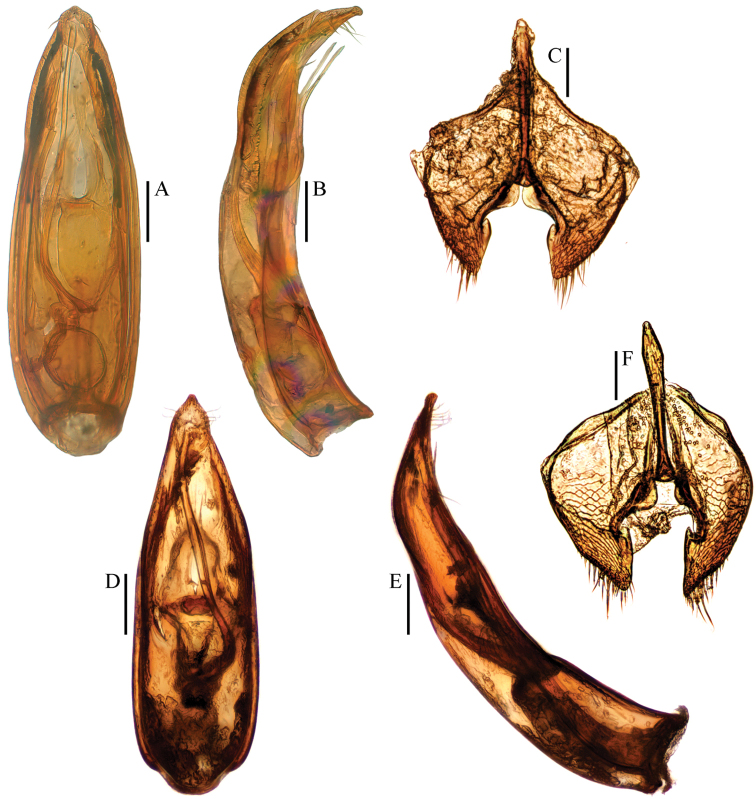
**A–C** Ptomaphagus (s. str.) kuntzeni Sokolowski, 1957 ♂ (holotype) **D–F** Ptomaphagus (s. str.) amamianus Nakane, 1963, syn. n. ♂ (paratype) **A, D** aedeagi (dorsal view) **B, E** aedeagi (lateral view) **C, F** genital segments (ventral view). Scale bars 0.1 mm.

**Figure 3.**
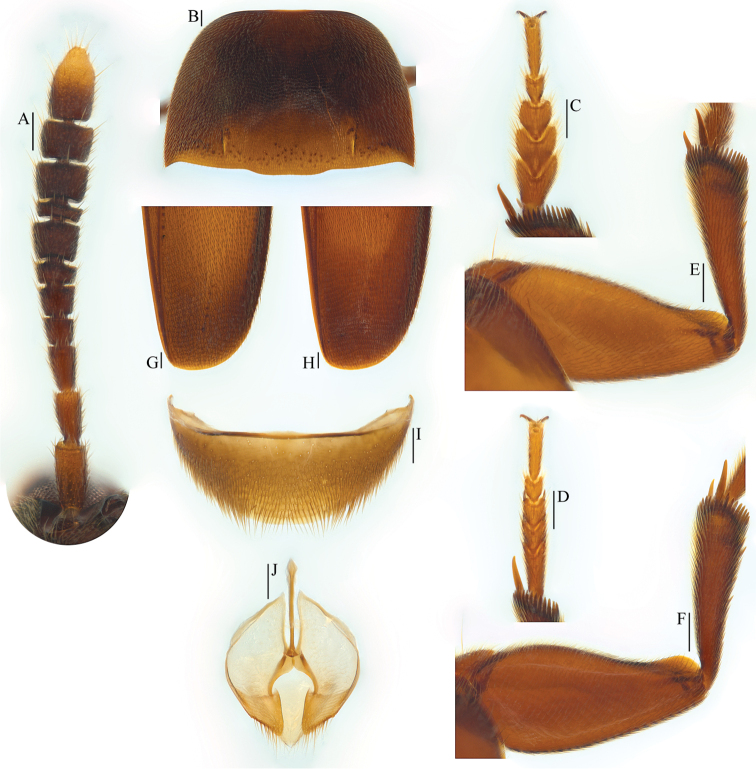
Ptomaphagus (s. str.) kuntzeni Sokolowski, 1957 (Amami Island). **A** antenna ♂ (dorsal view) **B** pronotum ♂ (dorsal view) **C** protarsus ♂ (dorsal view) **D** protarsus ♀ (dorsal view) **E** protibia and profemur ♂ (dorsal view) **F** protibia and profemur ♀ (dorsal view) **G** elytral apex ♂ (dorsoapical view) **H** elytral apex ♀ (dorsoapical view) **I** ventrite VIII ♂ (ventral view) **J** genital segment ♂ (ventral view). Scale bars 0.1 mm.

**Figure 4.**
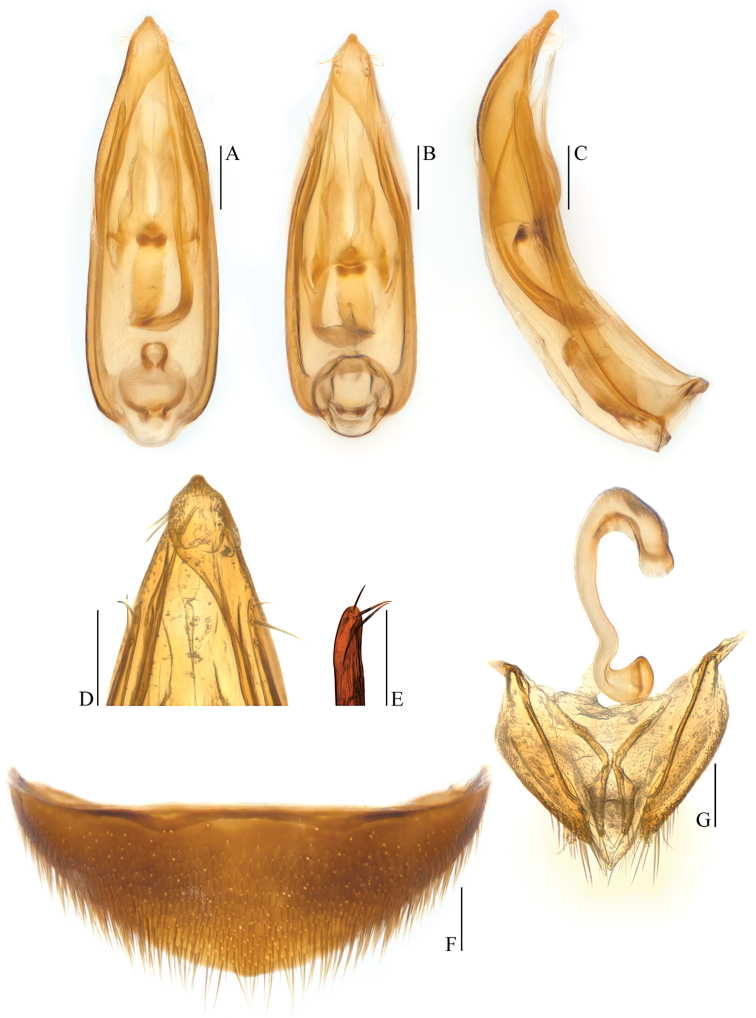
Ptomaphagus (s. str.) kuntzeni Sokolowski, 1957 (Amami Island). **A** aedeagus (dorsal view) **B** aedeagus (ventral view) **C** aedeagus (lateral view) **D** aedeagal apex (ventral view) **E** paramere apex (lateral view) **F** ventrite VIII ♀ (ventral view) **G** spermatheca and genital segment (ventral view). Scale bars 0.1 mm.

Figs 1A–C ; 2A–F ; 3A–J ; 4A–G

Ptomaphagus (s. str.) kuntzeni Sokolowski 1957: 140 (Ptomaphagus; type locality: [JAPAN] Hagi (? Landschaft Jamagutshi, Honshiu); ZMHB); Szymczakowski 1964: 63 (Ptomaphagus; female description; taxonomic remarks); Nishikawa 1983: 1 (Ptomaphagus (Ptomaphagus); in check-list); Harusawa and Yamamoto 2000: 242 (Ptomaphagus; distribution); Hayashi and Nishikawa 2010: 190 (Ptomaphagus; distribution); Perreau 2000: 363 (Ptomaphagus (s. str.); in catalogue); Perreau 2004: 178 (Ptomaphagus (Ptomaphagus); in catalogue); Nishikawa 2011: 100 (Ptomaphagus (Ptomaphagus); distribution; notes); Nishikawa et al. 2012: 274 (Ptomaphagus (Ptomaphagus); distribution); Perreau 2015: 249 (Ptomaphagus (Ptomaphagus); in catalogue).
Ptomaphagus (s. str.) amamianus Nakane 1963: 42 (Ptomaphagus; type locality: [JAPAN] Naze, Amami-Oshima); Hayashi 1969: 2 (Ptomaphagus; characteristic figures; distribution); Nishikawa 1983: 1 (Ptomaphagus (Ptomaphagus); in check-list); Perreau 1996: 284 (Ptomaphagus; distribution); Perreau 2000: 362 (Ptomaphagus (s. str.); in catalogue); Perreau 2004: 178 (Ptomaphagus (Ptomaphagus); in catalogue); Hayashi and Nishikawa 2010: 190 (Ptomaphagus; distribution); Perreau 2015: 249 (Ptomaphagus (Ptomaphagus); in catalogue). **Syn. n.**

#### Material examined.

**Type material. Holotype of *Ptomaphagus kuntzeni***: ♂, [JAPAN] Hagi [ca. 34°24'N, 131°23'E] / Hiller [R. leg., probably collected during 1872–1875 (Esaki 1935)] // Type // 59051 // Ptomaphagus / kuntzeni Type / det. K. Sokolowski (ZMHB). **Holotype of *Ptomaphagus amamianus***: ♂, HOLOTYPE // [JAPAN] NAZE [ca. 28°22'N, 129°29'E] / AMAMI[-ÔSHIMA] IS. / 4.V.1960 / T. Shibata [leg.] // Ptomaphagus / amamianus Nak. / Det. T. Nakane // NAKANE Coll. / SEHU JAPAN / 1999 // HOLOTYPE / Appended label by / N. Kobayashi / 2008 // 0000005731 / Sys. Ent / Hokkaido Univ. / Japan [SEHU] (HUM). **Allotype of *Ptomaphagus amamianus***: 1♀, ALLOTYPE // [JAPAN] NAZE [ca. 28°22'N, 129°29'E] / AMAMI[-ÔSHIMA] IS. / 4.V.1960 / T. Shibata [leg.] // Ptomaphagus / amamianus Nak. / Det. T. Nakane // NAKANE Coll. / SEHU JAPAN / 1999 // PARATYPE / Appended label by / N. Kobayashi / 2008 // 0000005732 / Sys. Ent / Hokkaido Univ. / Japan [SEHU] (HUM). **Paratypes of *Ptomaphagus amamianus***: 1♂, PARATYPE // [JAPAN] NAZE [ca. 28°22'N, 129°29'E], / AMAMI[-ÔSHIMA] IS. / 4.V.1960 / T. Shibata [leg.] // Ptomaphagus amamianus Nak. / Det. T. Nakane // MHNG / ENTO / 00003333 (MHNG); 1♀, same data as previous except: 00003334 (MHNG); 1♂, PARATYPE // [JAPAN] NAZE [ca. 28°22'N, 129°29'E] / AMAMI[-ÔSHIMA] IS. / 4.V.1960 / T. Shibata [leg.] // Ptomaphagus / amamianus Nak. / Det. T. Nakane // NAKANE Coll. / SEHU JAPAN / 1999 // PARATYPE / Appended label by / N. Kobayashi / 2008 // 0000005733 / Sys. Ent / Hokkaido Univ. / Japan [SEHU] (HUM); 1♀, [JAPAN] PARATYPE // // NAZE [ca. 28°22'N, 129°29'E] / AMAMI[-ÔSHIMA] IS. / 4.V.1960 / T. Shibata [leg.] // Ptomaphagus / amamianus Nak. / Det. T. Nakane // NAKANE Coll. / SEHU JAPAN / 1999 // PARATYPE / Appended label by / N. Kobayashi / 2008 // 0000005734 / Sys. Ent / Hokkaido Univ. / Japan [SEHU] (HUM); 1♂, PARATYPE // [JAPAN] NAZE [ca. 28°22'N, 129°29'E], AMAMI[-ÔSHIMA] IS. / 4.V.1960 / T. Shibata [leg.] // Ptomaphagus amamianus Nak. / Det. T. Nakane // 37–1 [Pl. 37, fig. 1 in Nakane 1963] // NAKANE Coll. / SEMU JAPAN / 1999 // PARATYPE / Appended label by / N. Kobayashi / 2008 // 0000005747 / Sys. Ent Hokkaido Univ. / Japan [SEHU] (HUM).

#### Additional material.

**JAPAN: Honshu**: 3♂♂1♀, Botanic Garden [ca. 38°15'N, 140°51'E] / Sendai, Miyagi [Pref.] / 1.IX.1966 / A. NARITA // Ptomaphagus / kuntzeni / Sokolowski / Det. T. Nakane (HUM); 2♂♂, Yoshi-ga-hira [ca. 37°26'N, 139°7'E] / Niigata Pref. / 14.VIII.1987 / leg. M. NISHIKAWA // Trap // alt. 600 m (CMNE); 1♂, ASAMIGAWAKEI / KOKU [ca. 37°13'N, 140°57'E] HIRONOMACHI / FUKUSHIMA [Pref.] 1990.VIII.2 / A. IZUMI leg. (CMNE); 1♀, Mt. TORINOKO-YAMA [ca. 36°41'N, 140°14'E] / 馬頭町 [Batô-machi], 栃木県 [Tochigi Pref.] / 30.APL.1996 / H. OHKAWA leg. // 14 (CMNE); 1♂, NAGAEDANI [ca. 36°34'N, 136°41'E] / KANAZAWA [Ishikawa Pref.] / 13.V.1948 / S. TAKABA [leg.] (CYHK); 1♂, Mt. TAKAO[-YAMA: ca. 36°27'N 136°45'E] / KAGA [=Ishikawa Pref.] / 2.VII.1961 / Y. Hayashi [leg.] // Ptomaphagus / kuntzeni / Sokolowski? / Det. Y. Hayashi, 1979 (CYHK); 1♂1♀, Tohbu [ca. 36°23'N, 138°21'E], Chiisagata / Nagano [Pref.], JAPAN / 1st, June, 1986 / legit. T. Abe (CMNE); 3♀♀, ISK. [= Ishikawa Pref.] Ishikawa Co. / Shiramine Vil. Shiramine [ca. 36°09'N, 136°37'E] / N. of Mt. Ohnadare / 13.VI–6.VIII.1988 // (rotten chicken trap) / 630 m, alt. / K. Katsura & / Y. Nishikawa leg. // ♀ (CJRZ); 4♂♂, ISK. [= Ishikawa Pref.] Ishikawa Co. / Shiramine Vil. Katarashi [ca. 36°09'N, 136°38'E] / NW. of Mt. Arigata-yama / 13.VI–6.VIII.1988 // (rotten chicken trap) / 580 m, alt. / K. Katsura & / Y. Nishikawa leg. // ♂ // Ptomaphagus kuntzeni SOKOLOWSKI / Det. K. Harusawa, 1993 (CJRZ); 1♂1♀, 石川県 [Ishikawa Pref.] 白山市 [Hakusan-shi] / 白山系 [Mts. Hakusan] / 猿壁堰堤 [Sarukabe-entei: ca. 36°06'N, 136°42'E] pit fall traps / 8月2–22日2002年 [2–22.VIII.2002] / 保科英人 [Hoshina, Hideto] 採集 [leg.] (CHHF); 1♂, GOZAISHI SPA [ca. 35°43'N, 138°21'E] / YAMANASHI [Pref.] / 12.VIII.1989 / TATEO ITO [leg.] (CYHK); 1♂, 1981–7–4 [= 4.VII.1981] / 日川林道上部 [Upper area of Hikawa-rindô: ca. 35°43'N, 138°50'E] // 大ボサツ [Mt. Daibosatsu-rei, Yamanashi Pref.] / 腐敗オサトラップ [decayed bait of carabid trap] // K. HAGA [leg.] (CMNE); 2♂♂, KAMIHIKAWA-RINDO [ca. 35°43'N, 138°50'E] / ([Mt.] DAIBOSATSU[-REI]) / Yamanashi Pref. / Aug. 29–30th 1982 / Y. Shibata leg. (CMNE); 2♂♂, 東京都西多摩郡 [Tokyo, Nishitama-gun] / 檜原村本宿 [Hinohara-mura, Motoshûku: ca. 35°43'N, 139°08'E] // Japan: Honshu / Motoshûku, / Hinohara Mura, / Tokyô To 12.IV.2008 / KAMEZAWA Hiromu leg. // KAMEZAWA Collection (CMNE); 1♂1♀, Mt. Takao[-san: ca. 35°37'N, 139°15'E] / Hachiôji, Tokyo / 16.X.1982 / M. Nishikawa leg. // Trap (CMNE); 4♂♂1♀, Mt. Mitsutoge-yama [ca. 35°32'N, 138°49'E] / 1200 m in alt., trap / Kawaguchiko-machi // Yamanashi Pref., C. / Japan, 19.VIII.1993 / M. Nishikawa leg. (CMNE); 1♂, Dohshigawa [Riv.: ca. 35°32'N, 139°06'E] / Aone, Tsukui Co. / Kanagawa [Pref.], JAPAN / 18th, May, 1986 / T. Abe & A. Sasai [leg.] (CMNE); 2♀♀, (Dôdaira [ca. 35°28'N, 139°10'E]) / E. Tanzawa / Kanagawa Pref. // 11.VII.1993 / leg. M. NISHIKAWA // carrion trap / alt. ca. 1000 m (CMNE); 1♂1♀, same data as previous except: 21.VIII.1993 (CMNE); 1♂, [JAPAN] Honshu, / Kanagawa Pref., Atsugi City, / Shimofurusawa [ca. 35°27'N, 139°19'E], / 23.III.2007. Bait trap. / Takuya FUKUZAWA leg. (CMNE); 1♀, same data as previous except: 22.III.2007 (CMNE); 2♀♀, Idenzawa [ca. 35°26'N, 138°58'E] / Nishitanzawa / Kanagawa [Pref.], Japan // 31.V.–6.VI.2006 / leg. T. Watanabe (CMNE); 1♂, Mt. OHYAMA [ca. 35°25'N, 139°14'E] / Kanagawa [Pref.], Japan / June 15th, 1974 / Coll. Y. Shibata (CMNE); 1♀, Hachikita-kôgen [ca. 35°24'N, 134°32'E] / Muraoka-machi / [A-1] baited-trap / Hyogo Pref. // W Honshu. Japan / 23.XI.2004 / Shigeru Yoshida leg. // 兵庫県村岡町 [Hyogo Pref., Muraoka-machi] / ハチ北高原 [Hachikita-kôgen] A-1 / bait-trap / 23.XI.2004 / 吉田 茂 [Yoshida Shigeru] 採集 [leg.] (CMNE); 1♂, Mt. Mikuni-yama [ca. 35°24'N, 138°54'E] / Nishitanzawa / Kanagawa [Pref.], Japan / 3–7.VIII.2003 / T. Watanabe leg. // Flight / Interception / Trap (CMNE); 1♀, (Near OHKURA [ca. 35°24'N, 139°10'E]) / Tanzawa [Mts.], Kanagawa [Pref.] / Apr. 29th, 1973 / Coll. Y. Shibata (CMNE); 3♂♂9♀♀, Kitanisawa 750 m alt. [ca. 35°19'N, 133°33'E] / Mitsukue, Kôfu / Hino-gun, Tottori // Pref., W Japan / 17.VI.2007; b.trap / Y. Fujitani leg. // 鳥取県江府町 [Tottori Pref., Kôfu-chô] / 御机 [Mitsukue] 木谷沢 [Kitanisawa] / bait-trap 750 m (CMNE); 1♂2♀♀, Takahachi [ca. 35°19'N, 138°43'E] on Mt. / Fuji (cola trap) / Shizuoka Pref. // Central Japan / 14.VII.–24.VIII.1996 / M. Nishikawa leg. (CMNE); 1♂, [Mt.] Fudohyama [ca. 35°19'N, 139°11'E] / Nakai-machi / Kanagawa [Pref.], Japan / 3–12.VI.2006 / T. Watanabe leg. (CMNE); 1♂, Hisagi [35°18'N, 139°34'E] / Zushi c. / Kanagawa pref. / 18–23.V.1983. / pit-fall Trap: No.32 / No. 2 (CMNE); 5♂♂12♀♀, Yakôdani [ca. 35°17'N, 134°18'E] / Chizu-machi / Yazu-gun, Tottori / Pref., W Honshu, Jpn / 3.VI.2007 / Y. Fujitani leg. // 鳥取県八頭郡 [Tottori Pref., Yazu-gun] / 智頭町八河谷 [Chizu-machi, Yakôdani] / bait-trap 750 m (CMNE); 6♂♂3♀♀, Near Nontaki waterfall [ca. 35°15'N, 134°08'E] / ca. 800 m in alt. / Aba-son, Tomata-gun / Okayama Pref., Japan / 8.IX.2002; trap / Y. Fujitani leg. (CMNE); 4♂♂2♀♀, Mt. Yamanori-yama [ca. 35°14'N, 133°49'E] / Chûka-son, Maniwa- / ca. 900 m in alt. / gun, Okayama Pref. // W Honshu, W Japan / 11.VII.2004; FIT / Y. Fujitani leg. (CMNE); 2♂♂2♀♀, Daruga-mine [ca. 35°12'N, 134°22'E] / Nishiawakura-son / 1,100 m in alt. / Aida-gun // Okayama Pref. / 7–20.VII.2006; FIT / Akihiko Watanabe leg. (CMNE); 1♂, Akazaigawa Val. [ca. 35°12'N, 134°31'E] / HYOGO Pref. / 12.V.1979 / Y. HAYASHI [leg.] // Ptomaphagus kuntzeni SOKOLOWSKI / Det. Y. HAYASHI, 2014 (CCBW); 1♂1♀, same data as previous except: 15.VII.1979 (MNHA); 1♀, nr. Mt. Suzuga-take [ca. 35°11'N, 136°27'E] / Fujiwara-chô, Mie Pref. / 3.V.2002 / Shiho Arai leg. // Ptomaphagus / kuntzeni / Sokolowski, 1957 / Det. M. Nishikawa, 2014 / #MNIC123909Ch1S (CMNE); 1♀, 千葉県 [Chiba Pref.] 君津市 [Kimitsu-shi] / 郷台畑 [Gôdaihata] / 猪の川 [Inokawa] / 滝の沢出合 [Takinosawa Deai: 35°11'N, 140°06'E] / malaise traps / 5月13–20日1997年 [13–20.V.1997] / 新田 N. [Nitta, N.] 採集 [leg.] (CHHF); 1♂2♀♀, MIZUHO T. [ca. 35°10'N, 135°22'E] / KYOTO, / 22.V.1984 / Y. HAYASHI [leg.] // trap // Ptomaphagus kuntzeni SOKOLOWSKI / Det. Y. HAYASHI, 2014 (1♂ in CCBW and 2♀♀ in CYHK); 1♂1♀, same data as previous except: 26.V.1984 (CCBW); 1♂3♀♀, same data as previous except: シズシ [Shizushi: 35°13'N, 135°20'E] // [bar code omitted] (CYHK; MNHA); 1♂, [Mt.] Takiyama [ca. 35°09'N, 134°09'E] / Nagi, Katsuta-gun / Okayama Pref. // W. Honshu, Japan / 9.V.2002 / Y. Fujitani leg. (CMNE); 8♂♂3♀♀, same data as previous except: 7.VII.2002 (CMNE); 3♂♂4♀♀, same data as previous except: 27.X.2002 (CYFO); 1♂, same data as previous except: 10.XI.2002 (CYFO); 1♀, same data as previous except: 23.XI.2002 (CYFO); 1♀, same data as previous except: 29.XII.2002 (CYFO); 19♂♂23♀♀, same data as previous except: 27.VI.2003 (CYFO); 1♀, JAPON KYOTO / Seryô-Tôgé Kyoto [ca. 35°09'N, 135°45'E] / 500–600 m 6.VIII.1980 / Cl. Besuchet [leg.] (MHNG); 1♀, SASAYAMA T. / 雨石山 [Mt. Amaishi-yama: ca. 35°07'N, 135°20'E] / HYOGO [Pref.], / 28.IV.1984 / Y. HAYASHI [leg.] (CYHK); 2♂♂3♀♀, nr. Rashomon-daiichi- / do Cave [ca. 34°56'N, 133°33'E], Niimi-shi. / 370 m in alt. / Okayama Pref. // W Japan (carrion trap) / 18.VIII.2001 / Y. Fujitani leg. (CMNE); 1♂1♀, Shiramizu [ca. 34°58'N, 134°15'E] / Mimasaka-shi / [carrion baited-trap] / Okayama Pref. // W Honshu, Japan / 7.V.2005 / Y. Fujitani leg. (CMNE); 4♂♂1♀, Mumyôdani [ca. 34°56'N, 133°27'E], ca. 350 m / Tetta-chô, Atetsu- / gun, Okayama Pref. // W Honshu, W. Japan / 25.IV.2004; b. trap / Y. Fujitani leg. (CMNE); 1♀, 箕面市下止々呂美 [Minô-shi, Shimotodoromi: ca. 34°52'N, 135°27'E] / (OSAKA pref.) / 4.VIII.1992 / leg. 齋藤琢己 [Saito Takumi] // Ptomaphagus / kuntzeni / SOKOLOWSKY / Det. Y. HAYASHI, 1993 (CYHK); 1♂4♀♀, Kanahira National / Forest, [ca. 34°47'N, 133°37'E] Takahashi- / [carrion baited-trap] / shi, Okayama Pref. // W Honshu, Japan / 16.IV.2006 / Y. Fujitani leg. (CMNE); 1♂, Anatoyama-jinja [ca. 34°39'N, 133°29'E] / 450 m alt., Kawakami / Okayama Pref. // Honshu, W Japan / 22.IV.2001; trap / Y. Fujitani leg. (CMNE); 1♀, same data as previous except: 8.IV.2001 (CMNE); 1♀, same data as previous except: 16.IV.2001 (CMNE); 1♂3♀♀, same data as previous except: 28.IV.2001 (CMNE); 1♀, Ômukai-rindô [ca. 34°29'N, 132°07'E] / Chôjabara, Yoshiwa / 880 m in alt. / Hatsukaichi-shi // Hiroshima Pref., W / Honshu, Jpn. 28.IV. / 2007, M. Tagami leg. // 広島県廿日市市 [Hiroshima Pref., Hatsukaichi-shi] / 吉和長者原大向林道 [Yoshiwa, Chôjabara, Ômukai-rindô] / FIT 880 m / 28.IV.2007 / 田上雅生 [Tagami Masao] 採集 [leg.] (CMNE); 1♂1♀, OSAKA Pr. Tondabayashi c. / Kannobi Kongô Colony [ca. 34°29'N, 135°35'E] / 12–26.IV.1994 / leg. K. Harusawa // pit fall trap: / rotten squid // Ptomaphagus kuntzeni Sokolowski (CMPR); 6♂♂3♀♀, Kasayama, ca. 50 m / Hagi-shi, / ca. 34°26'N, 131°24'E / Yamaguchi Pref. // W Honshu, W Japan / 29.VI.2002; trap / Y. Fujitani leg. (CMNE); 2♀♀, same data as previous except: 5.VII.2002 (CMNE); 2♂♂2♀♀, Mt. Makio[-san: ca. 34°23'N, 135°30'E] / OSAKA [Pref.] / 4.VIII.1983 / F. KIMURA [leg.] (CYHK); 1♂, [OSAKA Pr.] / Kawachinagano / 天見 [Amami], alt. 260 m / 6–14.X.1981 / K. Harusawa / Y. Nishikawa leg. // 出合 [Deai]~流谷 [Nagaredani] / 流谷八幡境内 [Nagaredani-hachiman shrine’s area: ca. 34°23'N, 135°35'E] / 腐肉 [carrion] Trap (牛 [beef]) // Ptomaphagus / kuntzeni / SOKOLOWSKI / Det. Y. HAYASHI, 1990 (CYHK); 1♂, Kamisuga [ca. 34°23'N, 136°22'E] / Ôdai-machi, Taki- / gun, Mie Pref. // Honshu, Japan / 22–9.V.2005 / Katsumi Akita leg. (CMNE); 3♂♂2♀♀, Kanoashikôchi [ca. 34°22'N, 131°57'E] / Muikaichi-machi / 450 m in alt. / Shimane Pref. // W Honshu, Japan / 10.VI.2005 / Y. Fujitani leg. // 六日市町 [Muikaichi-machi] / 鹿足河内 [Kanoashikôchi] (CMNE); 1♂, HASE [ca. 34°19'N, 135°48'E] / YAMATO [=Nara Pref.] / 5.VI.1966 / Y. HAYASHI [leg.] // Ptomaphagus / kuntzeni (CYHK); 1♂4♀♀, Nodani [ca. 34°17'N, 131°38'E], Tokuji-chô / Saba-gun / Yamaguchi Pref. // W Honshu, W Japan / 21.IV.2004; b. trap / Y. Fujitani leg. (CMNE); 2♀♀, mouth of Nakao-dô / Cave [ca. 34°16'N, 131°17'E]. Aokage / Shuhô, Yamaguchi // Pref., W Japan / 28.VII.–4.VIII.2002 / Y. Fujitani leg. (CMNE); 3♂♂5♀♀, Akiyoshidai [ca. 34°16'N, 131°18'E] / Shuho-machi / Yamaguchi Pref. // W Honshu, W Japan / 12.IV.2002; trap / Y. Fujitani leg. (CMNE); 1♂2♀♀, Mt. Nagano-yama [ca. 34°16'N, 131°52'E] / 1,010 m in alt. / b.trap / Kano-machi // Yamaguchi Pref. / Japan, 4.VI.2005 / Y. Fujitani leg. (CMNE); Japan. / G. Lewis. / B. M. 1926–369. // Chiuzenji [ca. 36°46'N, 139°28'E]. // Ptomaphagus / sibiricus Jean. / Jeannel det. // Ptomaphagus / kuntzeni ♀ / det. WANG C.-B., 2016 (BMNH). **Kyushu**: 1♂, Hata [ca. 33°48'N, 130°46'E] / Yahata-City [Fukuoka Pref.] / 3.V.1965 / coll. M. Ueda (HUM); 1♂, 福岡県 [Fukuoka Pref.] 添田町 [Soeda-cho] / 英彦山 [Mt. Hiko-san: ca. 33°29'N, 130°54'E] / 5月2日1983年 [2.V.1983] / 野村 S. [Nomura S.] 採集 [leg.] (CHHF); 1♀, (Kinsenji [ca. 32°58'N, 130°05'E], Mt. Tara[-dake]) / Nagasaki Pref. / Kyushu, JAPAN / 10.V.1983 / S. Imasaka leg. // *Ptomaphagus* (s. str.) / *kuntzeni* Sokolowski, 1957 / Det. M. Nishikawa, 2010 / ♀ (CMNE); 1♂, Mt. Gokabaru-dake [ca. 32°57'N, 130°04'E] / (1,058 m in alt.) / Nagasaki Pref. // NW Kyushu, Japan / 11–12.V.1991 / M. Nishikawa leg. // carrion trap / alt. ca. m (CMNE); 2♂♂, Unzen-bessho [ca. 32°44'N, 130°15'E] / Nagasaki Pref. / 1.VI.1983 / leg. S. Imasaka (CMNE); 1♂, Hagi [ca. 32°32'N, 130°56'E], Gokanoshô / Kumamoto Pref. / 18.VI.1984 / leg. S. Imasaka (CMNE); 1♀, Takao [ca. 32°28'N, 131°08'E], Shimofukura / Shiiba, Miyazaki Pref. / Kyushu, SW Japan / 26.VII.2015 / Takashi Watanabe leg. // Ptomaphagus (Ptomaphagus) / kuntzeni Sokolowski, 1957 / Det. M. Nishikawa, 2016 / MNIC124950Ch1S♀ (CMNE); 1♂, Inao dake [ca. 31°07'N, 130°53'E] / Tashiro cho / Kagoshima Pref. / 14.VII.1985 / T. TANABE leg. // alt. 300 m // Trap // Ptomaphagus / kuntzeni / Sokolowski / Det. T. Nakane (HUM); 1♂, same data as previous except: 575 m (HUM); 1♀, same data as previous except: 610 m (HUM). **Ryukyus: Amami-Ôshima Is.**: 1♀, 鹿児島県 [Kagoshima Pref.] / 奄美市 [Amami-shi] / 龍郷町 [Tatsugô-chô] / 芦徳 [Ashitoku: ca. 28°25'N, 129°36'E] / 05.V.2011 / 稲垣政志 [Inagaki Masashi] Leg. (CYHK); 1♂1♀, (JAPAN) Kagoshima pref., / Amami-Ohshima Is., / Honcha pass. [ca. 28°23'N, 129°33'E] / 11–14.IV.2007 / T. FUKUZAWA et al. [leg.] (CMNE); 1♀, NAZE [ca. 28°22'N, 129°29'E] / AMAMI[-ÔSHIMA] IS. / 4.V.1960 / T. Shibata [leg.] // Ptomaphagus / ohshimensis / NAKANE / Det. Y. HAYASHI, 1969 (CYHK); 2♀♀, same data as previous but no det. label (CYHK); 1♀, same data as previous except: // Ptomaphagus / amamianus / NAKANE / Det. Y. HAYASHI, 2009 // Collection of / Y. HAYASHI // [bar code omitted] (MNHA); 2♂♂4♀♀, Ôganeku / 28.3598N 129.3403E [ca. 28°21'N, 129°20'E]; 260 m alt. / Yamato-son / Amami-Ôshima Is., Ryukyus / Kagoshima Pref., SW Japan / 27.II.–2.III.2016; trap / M. Nishikawa leg. (CMNE); 4♀♀, nr. Hôkoku-jinja, ca. 350 m / 28.362331N 129.482009E [ca. 28°21'N, 129°28'E] / Amami-shi / Amami-Ôshima Is. / Kagoshima Pref., Ryukyus / SW Japan / 27.II.–2.III.2016; trap / M. Nishikawa leg. (CMNE); 3♂♂4♀♀, around Ôkawa Dam, ca. 28°20'N, 129°29'E, 130 m alt., Amami-shi, Amami-Ôshima Is. / Kagoshima Pref., Ryukyus / SW Japan / 27.II.–2.III.2016; trap / M. Nishikawa leg. (CMNE); 1♂, Toen, nr. Amami ForestPolis / 28.3176°N 129.3297°E [ca. 28°19'N, 129°19'E]; 180 m alt. / Yamato-son, Amami-Ôshima Is. / Ôshima-gun, Kagoshima Pref. / Ryukyus, SW Japan / 3–10.III.2015; bait trap / M. Nishikawa leg. (CMNE); 11♂♂18♀♀, (JAPAN) Kagoshima Pref., / Amami-Ôshima Is., / Yamato vil., Ôdana. 310 m alt. / N28°18'40.2 E128°55'32.2 / 5.III.2008. Carrion Baited trap. / Takuya FUKUZAWA leg. (CMNE); 8♂♂5♀♀, Naon-kengyôzôrin Forest [ca. 28°18'N, 129°20'E] / 名音県行造林 [Naon-kengyôzôrin Forest]; alt. 349 m / 28.3058°N, 129.3371°E / Yamato, Amami-Ôshima Is. // Ôshima-gun, Kagoshima Pr. / Ryukyus, SW Japan / 2–10.III.2015; bait trap / M. Nishikawa leg. // Ptomaphagus (Ptomaphagus) / amamianus Nakane, 1963 / Det. M. Nishikawa, 2015 (CMNE); 1♂, Mt. Yuwan[-dake: ca. 28°17'N, 129°19'E] / Amami-Ohshima Is / 29.III.1999 // Ptomaphagus amamianus NAKANE / Det. Y. HAYASHI, 2014 (CCBW); 1♀, same data as previous except: 28.IV.2000 / 江本健一 [Emoto, Ken’ichi] 採集 [leg.] // ♀ (CMNE); 1♀, same data as previous except: 7–12.V.2006 / T. Watanabe leg. (CMNE); 1♂3♀♀, same data as previous except: // 650–680 m, / 18–26.III.2010, baited / pitfall traps, forest, / Tomáš Lackner leg. (CJRZ); 9♂♂9♀♀, (JAPAN) Kagoshima Pref., / Amami-Ôshima Is., sumiyou Vil., santarou Touge Pass, 318 m alt. / N28°17'03.9 E129°25'16.2 / Carrion Baited Trap. 6.III.2008. / Takuya FUKUZAWA leg. (CMNE); 2♂♂1♀, same data as previous except: 12–14.IV.2007 (CMNE); 1♂1♀, same data as previous except: 13–16.IV.2007. FIT / T. FUKUZAWA et al. [leg.] (CMNE); 4♂♂2♀♀, 鹿児島県大島郡 [Kagoshima Pref., Ôshima-gun] / 宇検村 [Uken-son] ヤクガチョボシ山麓 [foot of Mt. Yakugachoboshi-yama: ca. 28°15'N, 129°21'E] // Japan; Ryukyu / foot of Yakugachoboshi-yama, / Uken Son, Oshima Gun, / Kagoshima Ken // (Is. Amami-o-shima) / 27.II.2004 / KAMEZAWA, Hiromu leg. (CMNE); 1♂, HATSUNO [ca. 28°15'N, 129°22'E] / Amami[-Ôshima] Is. / 31.III.1969 / H. NOMURA [leg.] // Ptomaphagus amamianus NAKANE / Det. Y. HAYASHI, 2014 (CCBW); 1♂, same data as previous except: 1.IV.1967 (CYHK); 1♀, same data as previous except: 3.IV.1967 (CYHK); 1♂, same data as previous except: 4.IV.1967 (CYHK); 2♂♂1♀, same data as previous except: 3.IV.1969 // K. TANIZAWA [leg.] (CCBW); 2♂♂1♀, same data as previous except: // Ptomaphagus / amamianus NAKANE / Det. Y. HAYASHI, 1993 (CMNE); 1♂1♀, same data as previous except: 31.III.1969 / H. NOMURA [leg.] (CYHK); 4♂♂, same data as previous except: 1.IV.1969 (CYHK); 4♂♂1♀, same data as previous except: 2.IV.1969 (CYHK); 1♂1♀, same data as previous except: 3.IV.1969 (CYHK); 7♂♂3♀♀, same data as previous except: K. Tanizawa [leg.] (CYHK); 1♀, same data as previous except: 5.V.1969 / Y. MAEDA [leg.] (CMNE); 1♀, Nishinakama [ca. 28°15'N, 129°24'E] / AMAMI[-ÔSHIMA] Isl. / 5.IV.1969 / K. TANIZAWA [leg.] (CYHK); 3♂♂3♀♀, nr. Yakugachi Tunnel / 28.2295N 129.3599E [ca. 28°13'N, 129°21'E]; 30 m alt. / Kamiyakugachi, Amami-shi / Amami-Ôshima Is., Ryukyus / Kagoshima Pref., SW Japan / 27.II.–2.III.2016; trap / M. Nishikawa leg. (CMNE); 1♀, (由井岳 [Mt. Yui-dake: ca. 28°11'N, 129°18'E]) / 瀬戸内 [Setouchi-chô] 奄美大島 [Amami-Ôshima Is.] / 25.IV.2000 / 江本健一 [Emoto Ken’ichi] 採集 [leg.] // ♀ (CMNE); 6♂♂, Aminoko-tôge Pass / 28.1893°N 129.3659°E [ca. 28°11'N, 129°21'E]; 350 m alt. / Setsuko, Setouchi-chô / Amami-Ôshima Is. / Ôshima-gun, Kagoshima Pref. / Ryukyus, SW Japan / 6–10.III.2015; bait trap / M. Nishikawa leg. (CMNE); 1♂3♀♀, 鹿児島県 [Kagoshima Pref.] / 大島郡 [Ôshima-gun] / 瀬戸内町 [Setouchi-chô: ca. 28°08'N, 129°18'E] / 05.V.2011 / 稲垣政志 [Inagaki, Masashi] Leg. (CYHK); 1♂, IKARI [unlocated] / AMAMI[-ÔSHIMA] IS. / 18.V.1960 / T. Shibata [leg.] (CYHK). **Ryukyus: Kume-jima Is.**: 3♀♀, Shirase-gawa [Riv.: ca. 26°20'N, 126°46'E] / Gushikawa-son / Kume-jima Is. // Ryukyus, SW Japan / 15–17.III.1998 / M. Maruyama leg. (CMNE). **Ryukyus: Okinawa-hontô Is.**: 1♂1♀, [Okinawa: JAPAN] / Uka-rindô [ca. 26°48'N, 128°14'E], alt. 250– / 300 m, Kunigami vill. / 8.II.2009 / Takashi Kurihara leg. // ベイトトラップ [bait trap] / 鳥の手羽元 [fowl wing sticks] // Ptomaphagus / kuntzeni? / Y. HAYASHI, 19 2014 (CYHK); 2♂♂, same data as previous except 7.II.2009 (CYHK); 2♂♂, Yona [ca. 26°45'N, 128°12'E] (Ohkuni 5) / Okinawa-jima Is. / Ryukyus // SW Japan / 25–27.IV.1996 / M. Nagano leg. (CMNE); 1♀, Nishime-dake [ca. 26°48'N, 128°16'E]. OKN [= Okinawa Pref.] / Date: 14.X.2002 / K. MASUMOTO leg. (CMNE); 2♀♀, [Okinawa: JAPAN] / Hama-rindô [ca. 26°43'N, 128°09'E], alt. 50– / 100 m, Kunigami vill. / 8.II.2009 / Takashi Kurihara leg. // ベイトトラップ [bait trap] / 鳥の手羽元 [fowl wing sticks] (CYHK); 4♂♂, (JAPAN, Ryukyus) / Okinawa pref. / Okinawa Is. / Kunigami Vil., Hiji [ca. 26°43'N, 128°10'E] / 13.III.2009. / Takuya FUKUZAWA leg. (CMNE); 1♂, 与那覇岳 [Mt. Yonaha-dake: ca. 26°42'N, 128°13'E] / 沖縄本島 [Okinawa-hontô Is.] / 14.iv.2000 / 保科英人 [Hoshina, Hideto leg.] / 腐肉トラップ [carrion trap] // 2 (CMNE); 7♂♂5♀♀, bank of Haneji-ô-kawa / Riv. [ca. 26°36'N, 128°01'E], 20–40 m in alt. / Nago-shi, Okinawa- // jima Is., Ryukyus / (c. trap) 18.IV.1993 / R. Yakita leg. (CMNE); 13♂♂7♀♀, same data as previous except: 8–18.IV.1993 (CMNE); 14♂♂9♀♀, (JAPAN, Ryukyus) / Okinawa Pref. / Okinawa Is. / Nago City, Genka. / 26°36'N, 128°04'E // 10–13.III.2009. / by flight Intercept Trap / T. Fukuzawa, T. Ishikawa & M. Kishi leg. (CMNE). **Ryukyus: Tokuno-shima Is.**: 1♂, (JAPAN) Kagoshima Pref. / Tokuno-shima Is., / Amagi Town, / Mt. Yamatogusuku-san [ca. 27°48'N, 128°55'E] / 29.II.–4.III.2008. (FIT) Takuya FUKUZAWA leg. (CMNE); 85♂♂67♀♀, (JAPAN) Kagoshima Pref. / Tokuno-shima Is., / Amagi Town, / Mt. Sankyô-dake, 185 m alt. / N27°46'13.6, E128°57'18.0 / Carrion Baited Trap. 4.III.2008. / Takuya FUKUZAWA leg. (CMNE); 4♂♂3♀♀, same data as previous except: 27.II.–4.III.2008. (FIT) (CMNE). **Sadoga-shima Is.**: 1♂, Mt. Donden-yama [ca. 38°07'N, 138°22'E] / Sado Is., Niigata / Pref., Honshu, Japan // 30.IV.–26.V. / 1990 / leg. M. NISHIKAWA // carrion trap / alt. ca. 200 m (CMNE). **Shikoku**: 1♀, [SHIKOKU] / Komenono [ca. 33°58'N, 132°51'E] / Matsuyama [Ehime Pref.] / 25.IV.1993 / Lizhen Li leg. (EUM); 1♂, (Ehime: Japan) / Aonamimachi [ca. 33°53'N, 132°51'E] / Matsuyama-shi / 24.V.2006 / Shôma Sejima leg. (EUM); 3♀♀, same data as previous except: 2.VI.2006 (EUM); 2♀♀, Minokoshi on Mt. / [ca. 33°51'N, 134°05'E] / Tsurugi-san / ca. 1400 m in alt. / Tokushima Pref. // Shikoku, Japan / 25.VII.2004; trap / Y. Fujitani leg. // 德島県東祖谷山村 [Tokushima Pref., Higashiiyayama-son] / 見の越 [Minokoshi] / bait-trap (CMNE); 2♂♂2♀♀, 瓶が森 [Mt. Kamegamori: ca. 33°47'N, 133°11'E] (alt. 1670 m) / 高知県吾川郡いの町 [Kôchi Pref., Agawa-gun, Ino-chô] / 寺川 [Terakawa] 10.V.–8.VIII.2009 / 吉田 正隆 [Yoshida Masataka] 採集 [leg.] // Mt. Kamegamori (alt. 1670 / m) Terakawa Ino-chô / Agawa-gun Kôchi-Pref. / 10.V.–8.VIII.2009 / Masataka YOSHIDA leg. // 鳥ガラトラップ [fowl bone trap] / 地中 [underground] (50 cm) (CMNE); 2♂♂1♀, [SHIKOKU] / Mt. Ishizuchi [ca. 33°44'N, 133°06'E] / Ehime Pref. / 27.VIII.1990 / alt. 800 m / S. Takano leg. (EUM); 2♂♂, same data as previous except: 13.IX.1990 / alt. 500 m (EUM); 1♂, EHIME [Pref.]; Japan / Mt. Saragamine [ca. 33°43'N, 132°53'E] / Shigenobu-Town / 22.IV.1999 / Tatsuya Kan leg. (EUM); 4♀♀, 156 JAPAN, Shikoku, / Jshizuchi [Ishizuchi] Mt. Nat. Park, / OMOGO Valley, [ca. 33°42'N, 133°05'E] 700 m, / S. & J. Peck leg. // 158 mega carrion traps, / mixed warm temperate / forest, 18–25.viii.1980 (CJRZ); 1♀, Nishidani [ca. 33°31'N, 132°57'E] / Yanadani, Ehime [Pref.] / 6–8.V.1994 / Sakai, Li, Aita leg. / (bait-trap) // No. 8 (EUM); 4♂♂2♀♀, (SHIKOKU) / Odamiyama [ca. 33°31'N, 132°53'E] / Ehime Pref. / 22.VIII.1984 / E. Yamamoto [leg.] (EUM); 1♂3♀♀, same data as previous except: 14.VIII.1984 / 山本栄治 [Yamamoto Eiji] 採集 [leg.] (EUM); 1♂2♀♀, Komi [ca. 33°30'N, 132°57'E] / Yanadani, Ehime [Pref.] / 6–8.V.1994 / Ohbayashi, Nishino, Okada [leg.] / (by bait-trap) // No. 2 (EUM); 1♂1♀, same data as previous except: No.1 (EUM); 4♂♂5♀♀, Tengu-Kôgen [ca. 33°28'N, 133°00'E] (alt. 1280 m) / Kumakôgen-chô Kamiuke- / na-gun Ehime-Pref. / 9.V.–25.VII.2010 (Fowl trap) / Masataka YOSHIDA leg. // 愛媛県上浮穴郡 [Ehime Pref., Kamiukena-gun] / 久万高原町天狗高原 [Kumakôgen-chô, Tengu-Kôgen] / (alt. 1280 m) 9.V.–25.VII. / 2010 (鶏ガラトラップ [chicken bone trap]) / 吉田正隆 [Yoshida Masataka] 採集 [leg.] (CYHK); 1♀, Nagano [ca. 33°27'N, 132°56'E] / Yusuhara-chô [Kôchi Pref.] / 1–2.X.1994 / Y. Utsunomiya [leg.] / (by bait-trap) // No. 9 (EUM). **Shimokoshiki-jima Is.**: 1♀, Mt. Odake [ca. 31°43'N, 129°44'E] / Is. Shimokoshiki[-jima] / Kagoshima-pref. / 20.VI.1982 / S. Imasaka leg. (CMNE).

#### Redescription.

*Male*. EBL: 3.5–4.4 mm (3.5 mm in holotype of *Ptomaphagus kuntzeni*). Length of different body parts: HL : AL : PL : ELL = 0.6 : 1.2 : 1.0 : 2.2 mm; width: HW : EW : PW : ELW = 1.0 : 0.1 : 1.6 : 1.6 mm. Proportion of antennomeres from base to tip in µm (length × width): 167 × 84, 118 × 68, 114 × 74, 67 × 80, 76 × 99, 53 × 114, 96 × 126, 42 × 128, 92 × 142, 97 × 138, 197 × 115.

Habitus (Fig. 1A, B) elongated oval, regularly convex and sublustrous. Well pigmented: mostly brown to dark brown, head darker; mouthparts, basal three or four antennomeres and apical half of ultimate antennomere, protarsi, and apical parts of meso- and metatarsi more or less paler. Dorsum continuously clothed with fine, recumbent, yellowish pubescence. Insertions of pubescence on dorsal surfaces of pronotum, elytra and femora aligned along transverse striolations; interspace between two striolations glabrous.

Head quite transverse, HW/HL = 1.6. Clypeofrontal suture absent. Clypeus with anterior margin slightly rounded. Compound eyes well developed, EW/HW = 0.1. Antennae (Fig. 3A) slender, AL/HW = 1.2; antennomere III as long as II; VI with length/width = 0.5; XI longest, elongated pear-shaped.

Pronotum (Fig. 3B) transverse, widest just before hind angles, PW/PL = 1.5. Sides gently arched, gradually narrowing from posterior to anterior; hind angles drawn out, acute and sharp. Posterior margin widely protruding in the middle part, distinctly emarginate near hind angles.

Elytra oval, widest near basal 2/7, ELL/EW = 1.4. Sides weakly arched, gradually narrowing from widest part to apices, which widely rounded (Fig. 3G). Sutural striae present. Metathoracic wings fully developed.

Prolegs robust, with basal three protarsomeres (Fig. 3C) moderately expanded: TW/BTW = 1.4. Protibiae (Fig. 3E) distinctly expanded towards apex. Profemora (Fig. 3E) broad. Mesotibiae arcuate, mesotarsi simply linear. Metatibiae slender and straight.

Abdominal ventrite VIII (Fig. 3I) round at posterior edge and with an inconspicuous median notch. Spiculum gastrale of genital segment (Figs 2C, F; 3J) with approx. 1/3 of length protruding beyond anterior edge of epipleurite IX.

Aedeagus stout and wide, with median lobe gradually narrowing towards an oblong apex and terminated by an obtusely rounded knob in dorsal view (Figs 2A, D; 4A); opening of genital orifice situated on dorsal surface, deeply cut inwards on left edge of median lobe at subapex. Ventral surface of the apex of the median lobe (Fig. 4B, D) inserted with 5 ventrally oriented setae on the left side and 4 ventrally oriented setae on the right side; parameres narrow, reaching almost to apical 1/5 of median lobe, each apex (Fig. 4E) with 2 lateral setae and 1 apical seta relatively shorter. In lateral view (Fig. 2B, E), median lobe distinctly thick, regularly bent ventrad and gradually tapering towards a subround apex. Endophallus with stylus quite slender, a cheliform complex below the base of stylus, and a circular complex at the basal region.

*Female*. Similar to male in general appearance (Fig. 1C), including elytral apices (Fig. 3H), but distinguished by the following characteristics: protarsi (Fig. 3D) simply linear; protibiae (Fig. 3F) narrower at apex; ventrite VIII (Fig. 4F) almost narrowly rounded at posterior edge; genital segment as shown in Fig. 4G; spermatheca (Fig. 4G) C-shaped in distal part, not coiled in proximal part.

#### Distribution.

China (Taiwan), Japan (Fig. 8), ?Myanmar.

#### Remarks.

Varying body size is not an unexpected intraspecific variation in *Ptomaphagus* species, and occurs in common European species such as Ptomaphagus (s. str.) sericatus (Chaudoir, 1845) and Ptomaphagus (s. str.) varicornis (Rosenhauer, 1847). Although the holotype and paratypes of Ptomaphagus (s. str.) amamianus (Fig. 1B, C) are larger than the holotype of Ptomaphagus (s. str.) kuntzeni (Fig. 1A), this does not prevent us from synonymising the two species because of their identically shaped aedeagus (Fig. 2A, B, D, E).

Ptomaphagus (s. str.) kuntzeni somewhat resembles Ptomaphagus (s. str.) sibiricus in general appearance, but the former has antennomere III as long as II (Fig. 3A), antennomere VI with length/width = 0.5 and elytral apices (Fig. 3G, H) widely rounded; while the latter has antennomere III a little shorter than II (Fig. 7A), antennomere VI with length/width = 0.4 and elytral apices (Fig. 7B) narrowly rounded.

It should be noticed that Szymczakowski (1964) described a single female specimen from Kambaiti, Myanmar (coll. Naturhistoriska Riksmuseet, Stockholm) as Ptomaphagus (s. str.) kuntzeni. We concur with the opinion of Nishikawa (2011) that the occurrence of Ptomaphagus (s. str.) kuntzeni in Myanmar is dubious because of the wide geographical gap and the discrepancies of the morphological description of Szymczakowski (1964) with specimens from Japan.

According to the present data, this species is one of the most widespread cholevines in Japan, known from Honshu, Shikoku, Kyushu and Ryukyus. However, we have not yet examined any specimens from the northernmost area of Honshu (above the 38^th^ parallel) or from the southern Kume-jima Island in Ryukyus (Fig. 8). The species is recorded herein from Sadoga-shima Island, Shimokoshiki-jima Island and Kume-jima Island for the first time. Incidentally, no *Ptomaphagus* species have been recorded from Hokkaido to date. Moreover, Perreau (1996) reported the species from Taiwan Island under the name Ptomaphagus (s. str.) amamianus. We will deal with this area in a next paper devoted to *Ptomaphagus* from Taiwan.

Collecting methods for the material examined mostly indicate a necrophagous association, such as traps baited with decaying animal matter.

### Ptomaphagus (s. str.) piccoloi sp. n.

Taxon classificationAnimaliaColeopteraLeiodidae

http://zoobank.org/AE951C06-DAB3-4FBF-8CE8-35BF2B77882D

**Figure 5.**
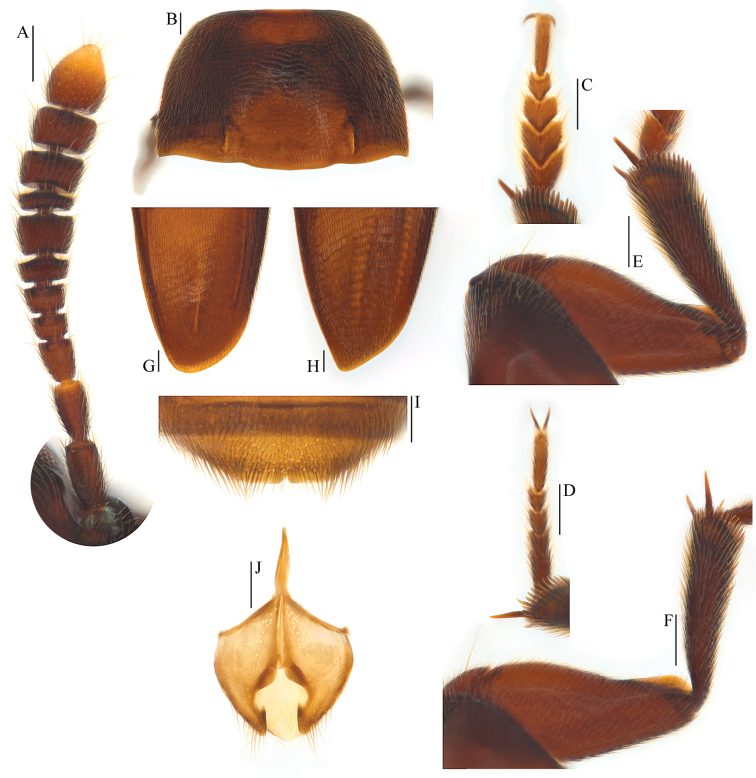
Ptomaphagus (s. str.) piccoloi sp. n. (paratype). **A** antenna ♂ (dorsal view) **B** pronotum ♂ (dorsal view) **C** protarsus ♂ (dorsal view) **D** protarsus ♀ (dorsal view) **E** protibia and profemur ♂ (dorsal view) **F** protibia and profemur ♀ (dorsal view) **G** elytral apex ♂ (dorsoapical view) **H** elytral apex ♀ (dorsoapical view) **I** ventrite VIII ♂ (ventral view) **J** genital segment ♂ (ventral view). Scale bars 0.1 mm.

**Figure 6.**
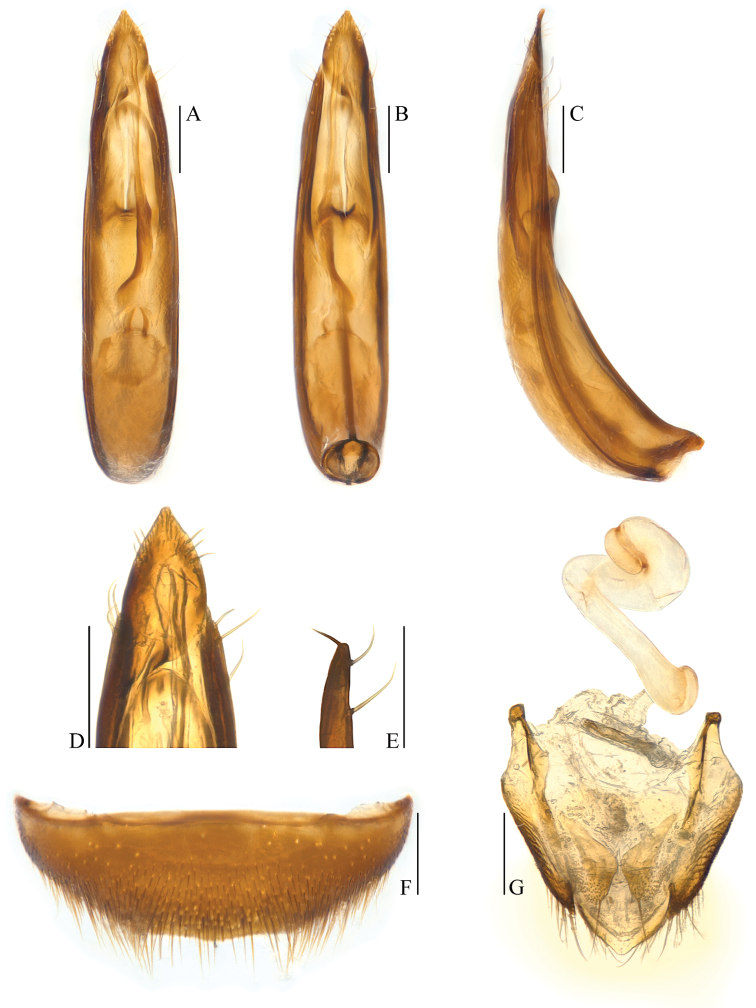
Ptomaphagus (s. str.) piccoloi sp. n. (paratype). **A** aedeagus (dorsal view) **B** aedeagus (ventral view) **C** aedeagus (lateral view) **D** aedeagal apex (ventral view) **E** paramere apex (lateral view) **F** ventrite VIII ♀ (ventral view) **G** spermatheca and genital segment (ventral view). Scale bars 0.1 mm.

Figs 1D, E ; 5A-J ; 6A-G

#### Type material.

**Holotype**: ♂, [JAPAN] Mt. Yamanori-yama [ca. 35°14'N, 133°49'E] / Chûka-son, Maniwa- / ca. 900 m in alt. / gun, Okayama Pref. // W Honshu, W Japan / 26.VI.2004; FIT / Y. Fujitani leg. // 5 (NSMT). **Paratypes**: 1♀, same data as holotype except: 2.VII.2004 (NSMT); 1♀, JAPAN, Ibaraki Pref. / Tsukuba City env. [ca. 36°04'N, 140°04'E] / 12.XI.2005 / P. Jałoszyński leg. // PTOMAPHAGUS (PTOMAPHAGUS) sibiricus JEANNEL, 1934 / det. H. Hoshina, 2006 (CPJA); 4♂♂6♀♀, Kurokura [ca. 35°25'N, 139°04'E] / ca. 400 m, Yamakita-machi / Ashigarakami-gun / Kanagawa Pref., C Japan // [trap: under stones at base of / debris slope] 29.IV.–14.V.2011 / M. Nishikawa leg. (2♂♂2♀♀ in CCBW, 1♂3♀♀ in CJRZ and 1♂1♀ in CMPR); 1♂, same data as previous except: 13.II.–18.III.2016 (CMNE); 1♂, [JAPAN] YAWATA [ca. 34°52'N, 135°42'E] / KYOTO [Pref.] / 2.II.1985 / T. ITO [leg.] // Ptomaphagus sibiricus JEANNEL / Det. Y. HAYASHI, 1985 (NMPC); 2♂♂1♀, [JAPAN] 岡山県美作市 [Okayama Pref., Mimasaka-shi] / 白水 [Shiramizu: ca. 34°58'N, 134°15'E] / bait-trap / 28.IV.2005 / 藤谷美文 [Fujitani Yoshifumi] 採集 [leg.] // Ptomaphagus sibiricus JEANNEL / Det. Y. HAYASHI, 2014 (1♂ in CMNE; 1♂ in CYHK; 1♀ in NMPC); 1♀, [JAPAN] KAKEYU [ca. 36°18'N, 138°08'E] / NAGANO. Pref. / 6.IV.1979. / Y. Hirano. leg. // ♀ (CMNE); 1♂, 東京都西多摩郡奥多摩町 [Tokyo, Nishitama-gun, Okutama-machi] / 日原 [Nippara] 一石山 [Mt. Isseki-zan] –人形山 [Mt. Ningyio-yama] // FIT (地上 [above the ground] 0.5–1 m) // Japan: Honshu [FIT No.2] / Mt. Isseki-zan–Mt. Ningyô- / yama (1040 m in alt.), Nippara / Okutama Machi, Tokyo / 35.858761, 139.036059 [ca. 35°51'N, 139°02'E] / 23.V.–8.VII.2015 / KAMEZAWA Hiromu leg. (CMNE); 1♂, same data as previous except: WFIT: 1100 m in alt. // 35.858796, 139.034128 (CMNE); 1♀, 東京都八王子市高尾山 [Tokyo, Hachiôji-shi, Mt. Takao-san: ca. 35°37'N, 139°14'E] // Japan: Honshu / Mt. Takao-san / (approx. 500 m in alt.), / Hachioji Shi, Tokyo To / 10.III.2007 / KAMEZAWA Hiromu leg. // KAMEZAWA Collection (CMNE); 1♀, same data as previous except: 14.II.2004 (CMNE); 1♂, [JAPAN] Mt. MASUGATAYAMA [ca. 35°36'N, 139°33'E] / KAWASAKI KANA / GAWA [Pref.] 1985.II.2 / Coll. A. IZUMI (CMNE); 1♂, [JAPAN] 宮ヶ瀬 [Miyagase] / 1-c // MIYAGASE [ca. 35°31'N, 139°13'E] / TANZAWA [Mts., Kanagawa Pref.] / 6.VI.1989 / H. HARADA [leg.] (CMNE); 1♂, same data as previous except: 2-c (CMNE); 1♂1♀, JAPAN Kanagawa-ken / Ashigarakami-gun / Yamakitamachi Kurokura [ca. 35°25'N, 139°04'E] / 29.iv.2011 alt. ca. 400 m / Ichiro Oshio leg. (CYFO); 1♂, [JAPAN] Mikuni-toge [ca. 35°24'N, 138°54'E] / Kanagawa [Pref.] / 7.VI.1970 / H. Takizawa [leg.] (CMNE); 1♀, [JAPAN] (Near BODAI [ca. 35°24'N, 139°11'E]) / Tanzawa [Mts.], Kanagawa [Pref.] / May 3rd 1973 / Coll. Y. Shibata (CMNE); 1♀, [JAPAN] 鳥取県 [Tottori Pref.] 西伯郡 [Saihaku-gun] / 大山町 [Daisen-chô] 三ノ沢 [Sannosawa: ca. 35°21'N, 133°32'E] / FIT alt 960 m / 6月7–23日2008年 [7–23.VI.2008] / 渡辺昭彦 [Watanabe, Akihiko] 採集 [leg.] (CYFO); 1♂, [JAPAN] 岡山県 [Okayama Pref.] 真庭市 [Maniwa-shi] / 蒜山 [Hiruzen] 蒜山大山S.L. [Hiruzen-Daisen Skyline road: ca. 35°19'N, 133°35'E] / 6月9–17日2007年 [9–17.VI.2007] / 渡辺昭彦 [Watanabe, Akihiko] 採集 [leg.] (CYFO); 1♂, [JAPAN] Jigokuana Cave [ca. 35°19'N, 135°27'E] / Mt. Chorosan / Wachi, KYOTO / 16.XI.1986 // Y. NISHIKAWA [leg.] // 仏主の地獄穴 [Hodosu-no-jigokuana Cave] / 16.XI.1986 (西川 [Nishikawa]) / Y. NISHIKAWA [leg.] (CYHK); 1♂, [JAPAN] OH-DOH [ca. 35°19'N, 139°19'E] 大堂 [Ôdô] (Tul.) / Mt. Koma-yama Ohiso town / Kanagawa Pref. / 23.III.2001 / Shiho ARAI leg. (CMNE); 1♀, JAPAN Kanagawa-ken / Kamakura-shi / Inamuragasaki [ca. 35°18'N, 139°31'E] / 2.i.2011 / Eimon Ueda leg. (CYFO); 1♀, Shiraishijizo-no-ana / Cave [ca. 35°14'N, 139°06'E], Hakoneyumoto / Kanagawa Pref. // C Japan, Ethanol trap / 7.IV.–18.V.1996 / M. Nishikawa leg. (CMNE); 1♀, same data as previous except: 22.VI.1996 (CMNE); 1♂, same data as previous except: 14.VIII.1996 (CMNE); 3♂♂2♀♀, same data as previous except: baited trap / 16.IV.–13.V.1995 (CMNE); 1♀, [JAPAN] Kanagawa Pref. / Odawara C. / Iriuda [ca. 35°14'N, 139°07'E] / 15 Oct.1995 / H. Miyatani Leg. (CMNE); 1♂, [JAPAN] YUGAWARA [ca. 35°09'N, 139°05'E] / HAKONE [Kanagawa Pref.] / 29.IV.1984 / Y. Hirano. leg. (CMNE); 2♀♀, [JAPAN] 岡山県 [Okayama Pref.] 新見市 [Niimi-shi] / 千屋ダム湖畔 [Chiya Dam lakeside: ca. 35°03'N, 133°27'E] / FIT alt 460 m / 6月7–23日2008年 [7–23.VI.2008] / 渡辺昭彦 [Watanabe, Akihiko] 採集 [leg.] (CYFO); 1♂, (Kusama [ca. 34°56'N, 133°32'E], Niimi-shi) / Okayama Pref., Honshu / Japan. May 7th, 1997 / Coll. Y. Watanabe (CMNE); 1♂, nr. Rashomon-daiichi- / dô Cave [ca. 34°56'N, 133°33'E], Niimi-shi. / 370 m in alt. / Okayama Pref. // W Japan (carrion trap) / 18.VIII.2001 / Y. Fujitani leg. (CMNE); 1♀, same data as previous except: 27.III.2005 (CMNE); 1♀, Mt. Arato-yama [ca. 34°55'N, 133°22'E] / ca. 600 m, Tetta-chô / litter: sieve / Atetsu-gun // Okayama Pref. / W. Japan, 25.V.2003 / Y. Fujitani leg. (CMNE); 1♂, [JAPAN] 岡山県阿哲郡 [Okayama Pref., Atetsu-gun] / 哲多町 [Tetta-chô] 無明谷 [Mumyôdani: ca. 34°53'N, 133°27'E] / bait-trap // 25.IV.2004 / 藤谷美文 [Fujitani Yoshifumi] 採集 [leg.] (CMNE); 1♂, [JAPAN] MINOO [ca. 34°50'N, 135°28'E] / Osaka / 5.iv.1961 / Y. Kimura [leg.] // Ptomaphagus / sibiricus / Det. Y. HAYASHI, 19 (CYHK); 1♀, 28.v.2007 / [JAPAN] 広島県神石高原町 [Hiroshima Pref., Jinsekikôgen-chô] / 高光 [Takamitsu: ca. 34°48'N, 133°10'E] リターより [from litter] / 妹尾鈴香 [Senoo Rinka leg.] (CYFO); 1♂, [JAPAN] (Ôdaru-onsen [ca. 34°47'N, 138°56'E]) / Shizuoka [Pref.], Honshu / March 22nd,1983 / Coll. Y. Watanabe (CMNE); 1♀, [JAPAN] Mt. AOMINE [= Mt. Aonomine-san: ca. 34°24'N, 136°49'E] / TOBA, MIE [Pref.] / 17.VIII.1988 / T. ITO [leg.] (CYHK); 1♀, [JAPAN] Mt. Hiko[-san: ca. 33°29'N, 130°54'E] / Hukuoka [= Fukuoka Pref.] / 17–19.V.1967 / H. Takizawa [leg.] (CMNE); 2♀♀, [JAPAN] Ohsé-no-ko-ana / Cave [ca. 32°16'N, 130°36'E], Ohsé / Kuma-mura / Kumamoto Pref // SW JAPAN / 26.V.1998 / S. Uéno & S. Arai leg. [recorded as Ptomaphagus (Ptomaphagus) sp. in Nishikawa 1995] (CMNE); 1♂, [JAPAN] 屋代島 [Yashiro-jima Is.]. 源明峠 [Genmei-tôge: ca. 33°53'N, 132°14'E] / 山口県周防大島町 [Yamaguchi Pref., Suo-Ôshima-chô] / 27.VI.2005 / Leg. 伴一利 [Ban Kazutoshi] (CMNE).

#### Description.

*Male*. EBL: 2.7–3.0 mm (2.7 mm in holotype). Length of different body parts: HL : AL : PL : ELL = 0.5 : 0.8 : 0.8 : 1.7 mm; width: HW : EW : PW : ELW = 0.7 : 0.1 : 1.2: 1.3 mm. Proportion of antennomeres from base to tip in µm (length × width): 116 × 61, 91 × 56, 70 × 61, 40 × 66, 43 × 76, 44 × 95, 67 × 102, 32 × 107, 59 × 112, 68 × 113, 121 × 104.

Habitus (Fig. 1D) elongated oval, regularly convex and sublustrous. Well pigmented: mostly brown; mouthparts, apical half of ultimate antennomere, protarsi, and apical parts of meso- and metatarsi more or less paler. Dorsum continuously clothed with fine, recumbent, yellowish pubescence. Insertions of pubescence on dorsal surfaces of pronotum, elytra and femora aligned along transverse striolations; interspace between two striolations glabrous.

Head transverse, HW/HL = 1.5. Clypeofrontal suture absent. Clypeus with anterior margin slightly rounded. Compound eyes small, EW/HW = 0.1. Antennae (Fig. 5A) slender, AL/HW = 1.1; antennomere III a little shorter than II; VI with length/width = 0.5; XI pear-shaped.

Pronotum (Fig. 5B) transverse, widest just before hind angles, PW/PL = 1.6. Sides gently arched, gradually narrowing from posterior to anterior; hind angles slightly drawn out and bluntly rounded. Posterior margin widely protruding in the middle part, distinctly emarginate near hind angles.

Elytra oval, widest at approx. basal 1/4, ELL/EW = 1.35. Sides weakly arched, gradually narrowing from widest part to apices, which narrowly rounded (Fig. 5G). Sutural striae present. Metathoracic wings fully developed.

Prolegs robust, with basal three protarsomeres (Fig. 5C) moderately expanded: TW/BTW = 1.5. Protibiae (Fig. 5E) distinctly expanded towards apex. Profemora broad. Mesotibiae slightly arcuate, mesotarsi simply linear. Metatibiae much thick and straight.

Abdominal ventrite VIII (Fig. 5I) almost round at posterior edge and with a distinct median notch. Spiculum gastrale of genital segment (Fig. 5J) with nearly 1/2 of length protruding beyond anterior edge of epipleurite IX.

Aedeagus small, slender and narrow, with median lobe gradually narrowing towards a leaf-shaped apex and terminated by an obtusely rounded knob in dorsal view (Fig. 6A); opening of genital orifice situated on dorsal surface, deeply cut inwards on left edge of median lobe at subapex. Ventral surface of the apex of the median lobe (Figs 6B, D) inserted with 4 ventrally oriented setae on the left side and 5 ventrally oriented setae on the right side; parameres narrow, reaching almost apical 1/8 of median lobe, each apex (Fig. 6E) with 2 lateral setae and 1 apical seta relatively shorter. In lateral view (Fig. 6C), median lobe thin, bent in basal half and almost straight in apical half, gradually tapering apically. Endophallus with stylus quite slender, a cheliform complex just below the base of stylus, and a circular complex at the basal region.

*Female*. Similar to male in general appearance (Fig. 1E), but distinguished by the following characteristics: protarsi (Fig. 5D) simply linear; protibiae (Fig. 5F) narrower; elytral apices (Fig. 5H) acuminate; ventrite VIII (Fig. 6F) slightly protruded in median of posterior margin; genital segment as shown in Fig. 6G; spermatheca (Fig. 6G) sinuous or coiled in distal part, not coiled in proximal part.

#### Distribution.

Japan (Fig. 9).

#### Etymology.

The specific epithet is from the name of “Piccolo”, a fictional character in the *Dragon Ball* manga series created by Akira Toriyama, which also has an Italian origin meaning of “small” that refers to the small body size of this new species.

#### Remarks.

**Figure 7.**
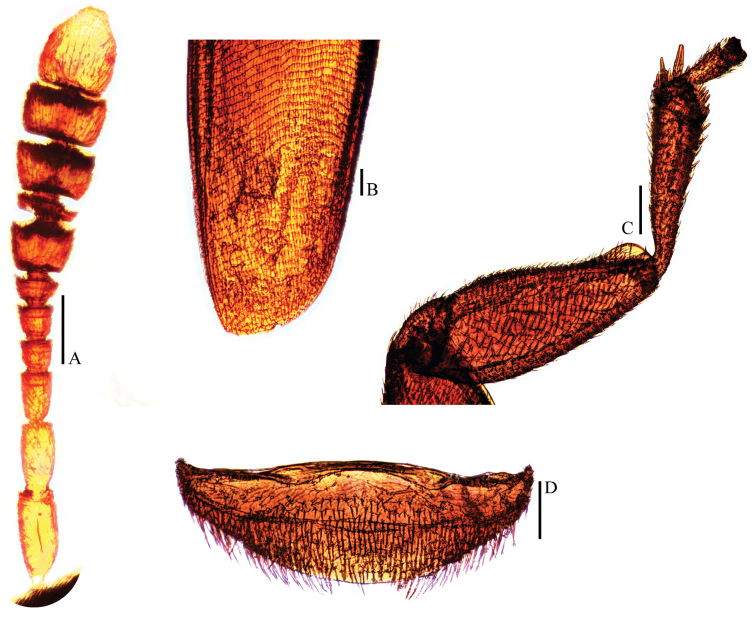
Ptomaphagus (s. str.) sibiricus Jeannel, 1934 ♀ (holotype). **A** antenna (dorsal view) **B** elytral apex (dorsoapical view) **C** protibia and profemur (ventral view) **D** ventrite VIII (ventral view). Scale bars 0.1 mm.

Several examined specimens of this tiny species had been previously identified as Ptomaphagus (s. str.) sibiricus (maybe more specimens are deposited in different Japanese researchers’ collections); however, it is conspicuously different to the holotype of Ptomaphagus (s. str.) sibiricus. The new species is smaller, with metatibiae much thicker (Fig. 1D, E) and female elytral apices acuminate (Fig. 5H); while Ptomaphagus (s. str.) sibiricus is larger, with metatibiae slender (Fig. 1F) and female elytral apices narrowly rounded (Fig. 7B).

Interestingly, the new species has the thickest metatibiae and most apparent sexual dimorphism on the elytral apices that we have encountered in any *Ptomaphagus* from East Asia and adjacent areas.

The new species has hitherto been known from Honshu and Kyushu (Hisamatsu and Hayashi 1985, for example), Japan, under the name Ptomaphagus (s. str.) sibiricus. Despite a long history of studies, it has not yet been recorded from Shikoku; also we have not examined any specimens of this new species from northern Honshu, above the 37^th^ parallel (Fig. 9). In these two areas, the new species can be regarded as at least rare or more probably completely absent. Specimens from Yashiro-jima Island in the Setonaikai Inland Sea were previously reported (as *Ptomaphagus sibiricus*) in Tanaka and Ban (2006).

As indicated in the material examined, Ptomaphagus (s. str.) piccoloi sp. n. has been collected from various habitats such as caves, under stones at the base of debris slopes and in litter layers.

Collecting methods for the material mostly indicate a necrophagous association, such as traps baited with decaying animal matter.

### Ptomaphagus (s. str.) sibiricus

Taxon classificationAnimaliaColeopteraLeiodidae

Jeannel, 1934

Figs 1F ; 7A–D

Ptomaphagus(s. str.)sibiricus Jeannel 1934: 165 (Ptomaphagus (s. str.); type locality: [RUSSIA, Far East] Wladiwostok; SDEI); Jeannel 1936: 72, 84 (Ptomaphagus (s. str.); in key; distribution); Nishikawa 1983: 1 (Ptomaphagus (Ptomaphagus); in check-list); Perreau 2000: 364 (Ptomaphagus (s. str.); in catalogue); Perreau 2004: 178 (Ptomaphagus (Ptomaphagus); in catalogue); Zinchenko and Lyubechanskii 2008: 340 (Ptomaphagus; distribution); Perreau 2015: 249 (Ptomaphagus (Ptomaphagus); in catalogue).

#### Material examined.

**Type material. Holotype**: ♀, [RUSSIA, Far East] Wladiwostok [ca. 43°10'N, 132°00'E] // Reitter // Coll. Koltze // Pt. variicornis / Rosenh. // Ptomaphagus sibiricus Jeann. / type / R. Jeannel det. // DEI Müncheberg / Col – 07069 (SDEI).

#### Distribution.

Russia (Far East).

#### Remarks.

Jeannel (1936) thought that Ptomaphagus (s. str.) sibiricus is also distributed in Japan based on a single female specimen from Chiuzenji (deposited in BMNH). So far, we have not seen any specimens from Japan identical with the holotype of Ptomaphagus (s. str.) sibiricus, and examined specimens previously identified as Ptomaphagus (s. str.) sibiricus actually belong to Ptomaphagus (s. str.) piccoloi sp. n. After examining this Japanese female specimen, labeled “Japan. / G. Lewis. / B. M. 1926–369. // Chiuzenji [ca. 36°46'N, 139°28'E]. // Ptomaphagus / sibiricus Jean. / Jeannel det.”, we found it actually belongs to Ptomaphagus (s. str.) kuntzeni, so Ptomaphagus (s. str.) sibiricus is absent in Japan.

Unfortunately, the spermatheca and genital segment of the holotype of Ptomaphagus (s. str.) sibiricus are missing. This species will be described in another paper after examining more specimens from the Russian Far East and the Korean Peninsula.

**Figure 8.**
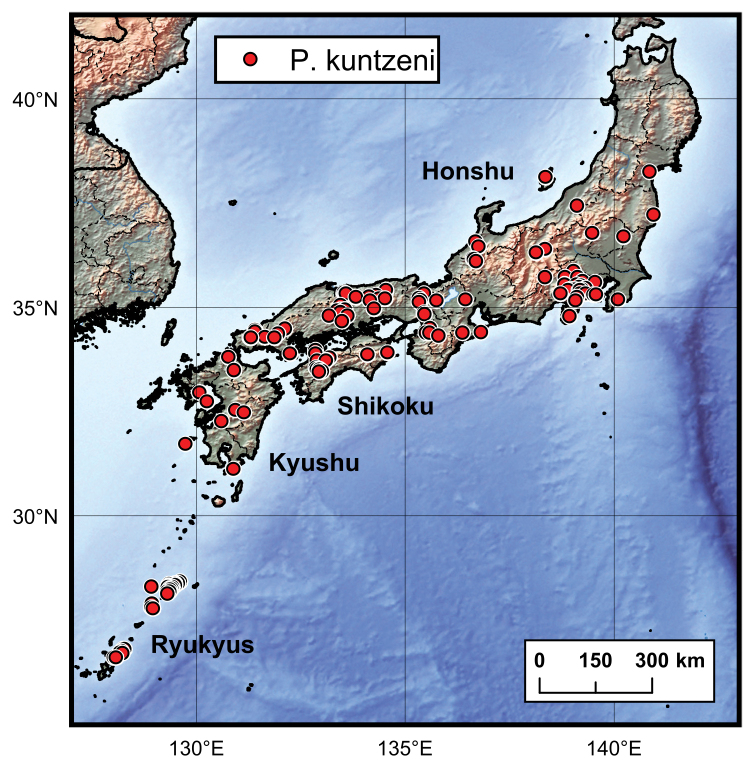
Distribution map of Ptomaphagus (s. str.) kuntzeni Sokolowski, 1957 in Japan.

**Figure 9.**
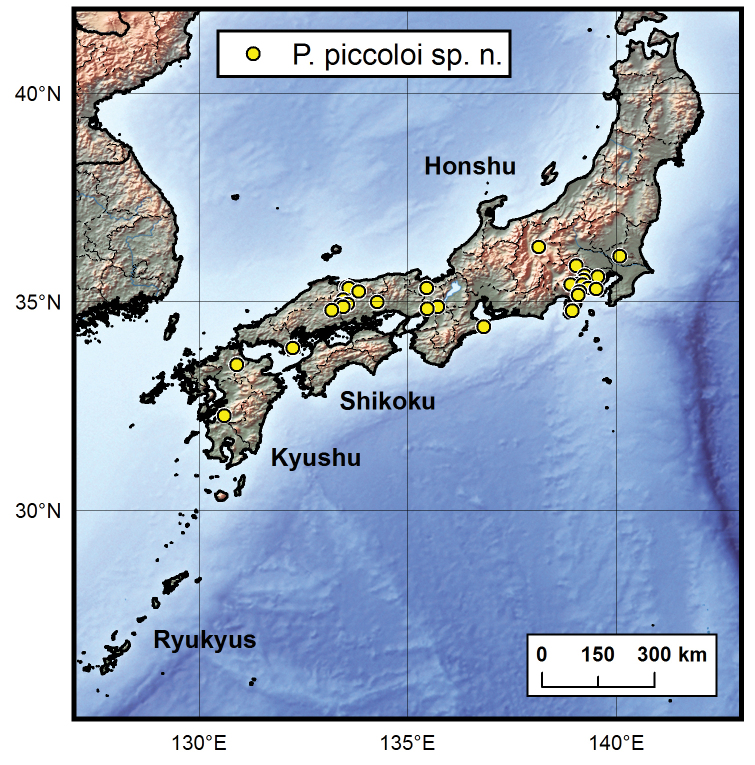
Distribution map of Ptomaphagus (s. str.) piccoloi sp. n. in Japan.

## Supplementary Material

XML Treatment for Ptomaphagus

XML Treatment for Ptomaphagus

XML Treatment for Ptomaphagus (s. str.) kuntzeni

XML Treatment for Ptomaphagus (s. str.) piccoloi

XML Treatment for Ptomaphagus (s. str.) sibiricus
